# Neuroprotective effect of resveratrol in diabetic cerebral ischemic-reperfused rats through regulation of inflammatory and apoptotic events

**DOI:** 10.1186/1758-5996-6-88

**Published:** 2014-08-17

**Authors:** Hoda E Mohamed, Sahar E El-Swefy, Rehab A Hasan, Ahmed A Hasan

**Affiliations:** Department of Biochemistry, Faculty of Pharmacy, Zagazig University, Zagazig, Egypt; Department of Histology, Faculty of Medicine, Al-Azhar University, Cairo, Egypt

**Keywords:** Diabetes, Ischemic-reperfusion, COX2, Resveratrol and apoptosis

## Abstract

**Background:**

Diabetes and cerebral ischemic-reperfusion are among the most common causes of neurological complications in Egypt. The prevalence of diabetes in Egypt is high and it can be considered as a major clinical and public health problem.

**Methods:**

Blood glucose, lipid profile, oxidative stress makers (cerebral MDA & GSH), cerebral interleukin-4 (IL-4) level and cerebral cyclooxygenase-2 (COX-2) gene expression were measured in male albino rats weighing 200 ± 20 g. The rats were divided into five groups, normal control group, diabetic group (diabetes was induced by single dose of streptozotocin [STZ]), diabetic cerebral ischemic-reperfused group, two treated groups (diabetic and diabetic ischemic-reperfused), both groups treated with resveratrol. Histological study was done using H&E, AgNOR and cresyl violet stains. Immunohistochemistry for Bax and COX-2 was done with morphometric study.

**Results:**

Diabetic and diabetic cerebral ischemic- reperfused rats showed significant increase in serum glucose level, serum TAG, serum LDL-C, atherogenic index, cerebral MDA and upregulation of COX-2 gene expression. These groups showed significant decrease in serum HDL, cerebral IL-4 and depletion of cerebral GSH when compared to normal control rats. Treating these groups with resveratrol resulted in significant decrease in serum glucose level, serum TAG, TC, serum LDL-C, atherogenic index, cerebral MDA and downregulation of COX-2 gene expression. The results of COX-2 gene expression were confirmed by COX-2 immunohistochemistry. Also, significant increase in serum HDL, cerebral IL-4 and cerebral GSH contents could be observed in these treated groups as compared to normal control group. Cerebral apoptotic index and optical density of Bax reaction revealed significant increase in diabetic and diabetic cerebral ischemic-reperfused rats while treatment of these groups with resveratrol resulted in significant decrease in cerebral apoptotic index and optical density of Bax reaction. These apoptotic results were confirmed with AgNOR and cresyl violet stains.

**Conclusion:**

The results of this research suggest that upregulation of cerebral COX-2 gene along with the decrease in cerebral IL-4 and enhanced cerebral apoptosis is critically involved in cerebral damage associated with diabetes and cerebral ischemic-reperfusion. Resveratrol can ameliorate these effects and has promising neuroprotective effect in diabetic-induced cerebral complications.

## Background

Diabetes mellitus is a chronic metabolic disorder, characterized by disturbed glucose metabolism due to an absolute or relative insulin deficiency [1]. The prevalence of diabetes in Egypt is high and it can be considered as a major clinical and public health problem [2]. Neurological complications are among the central problems in diabetes mellitus. Over 60% of individuals with diabetes are affected by neurological disorders [3]. Diabetic neuropathy is attributed to chronic hyperglycemia which may induce damage to nerve cells and decrease neurovascular flow especially during neuronal ischemia [4]. Cerebral ischemia is a leading cause of death and disability worldwide and diabetes is a risk factor for ischemic cerebrovascular diseases [5]. Reperfusion following cerebral ischemia leads to the generation of pro-oxidant species which cause neuronal damage by acting directly on macromolecules, including proteins, lipids and DNA, or indirectly by interfering with cell signaling pathways and gene expression regulation [6]. Furthermore, several mechanisms are responsible for diabetic neuropathy including dyslipidemia, inflammation and apoptosis [4, 7, 8] *.*

The most proposed molecular mechanism by which hyperglycemia induces complications in diabetes is increased oxidative stress [9]*.* Oxidative stress is a relative overload of oxidants caused by increased free radical production and/or decreased antioxidant defense systems. Increased free radical production exerts toxic effects on membrane phospholipids, resulting in formation of toxic products such as MDA [1, 10] *.*

Diabetes is usually associated with inflammation [8] *.* When excess glucose is shunted through alternative metabolic pathways, this leads to increase in TGF-β1 and NF-κB (inflammatory mediators). COX-2 is an important enzyme that is upregulated by NF-κB, which is observed in peripheral nerves and vascular tissues in experimental diabetes. Pharmacological blockade or gene ablation of COX-2 prevents diabetes-induced changes in peripheral nerves [4]*.* Also, COX-2 enzyme is responsible for the production of prostaglandins, a family of powerful inflammatory mediators produced by activated microglia in the neuroinflammatory/neurodegenerative diseases, and not surprisingly, COX-2 has been considered a major therapeutic target [11]*.* IL-4 has been demonstrated to have anti-inflammatory activities specially on activated microglia, including inhibition of the expression of TNF-α, as well other pro-inflammatory cytokines [12]*.* Apoptosis, programmed cell death, can contribute to a variety of disease states in the nervous system such as diabetes, ischemia and Alzheimer’s disease [13]*.*

Resveratrol, naturally occurring polyphenol, is found in high concentration in the skin and seeds of grapes, peanuts and ground nuts and has been reported to have several biological effects, including a potent antioxidative effect via preventing lipid peroxidation, cardioprotective, anticancer, and anti-inflammatory activity attributed to COX-2 inhibition [10, 14]*.* Moreover, resveratrol may be helpful in preventing and treating some metabolic diseases, including diabetes through reduction of blood glucose, preservation of cells, and improvement in insulin action [14]*.* In addition, resveratrol can reduce the oxidative stress produced in STZ-diabetic rats. [15]*.* It has been reported that resveratrol administration to the hypercholesterimic rats attenuated the increase in serum lipid profile [16]*.*

The present study was designed to clarify the adverse effects of diabetes on cerebral outcomes, evaluate the effect of resveratrol on modulating cerebral complications in diabetic and diabetic cerebral ischemic-reperfused rats and investigate the histological changes in cerebral tissue of diabetic, diabetic cerebral ischemic-reperfused and treated rats.

## Material and method

### Animals

Adult male albino rats (200 ± 20 g), purchased from The Egyptian Organization for Biological Products and Vaccines (Cairo, Egypt), were housed in stainless steel cages at room temperature (25 ± 2°C) and humidity of 65-69% and maintained on 12 hours light/dark cycle. Rats were fed on rodent chow (El-Nassr Pharmaceuticals. Co., Egypt). This protocol was approved by the Animal Care and Use Committee of Biochemistry Department, Faculty of Pharmacy, Zagazig University, Egypt.

### Experimental design

#### Induction of diabetes

One week after acclimatization, rats were fasted overnight. Diabetes was induced by intraperitoneal injection of a single dose of freshly prepared solution of streptozotocin (STZ) (45 mg/kg body weight) (Sigma Chemical Co. St. Louis, USA.) dissolved in 0.5 ml of 0.01 M cold sodium citrate buffer, pH = 4.5. One week later, blood samples were collected and processed for blood glucose determination. Rats which achieved fasting serum glucose level ≥200 mg/dl were considered diabetic and selected for this study [17]*.*

About 20% morbidity rate was shown during induction of diabetes and about 10% of animals were resistant and did not achieve the required serum glucose level. Rats were divided into two groups of 20 animals each. The first group: diabetic rats received equal amount of vehicle (distilled water with 3-5% w/v gum acacia), this group served as diabetic control (D) group. The second group: diabetic rats treated with resveratrol. *Resveratrol (MegaResveratrol®)* was supplied from Mega Resveratrol and Candlewood Stars Inc., USA, and given orally in a dose level 20 mg/kg body weight [18, 19] through the gavage tube once daily for six weeks prior to the induction of cerebral ischemic-reperfusion. Due to the limited water solubility of this drug, 3-5% w/v gum acacia was used as a suspending agent.

Another group of normal rats received equal amount of vehicle (distilled water with 3-5% w/v gum acacia) represents the normal control (N) group.

At the end of the treatment period, the rats were fasted overnight and the previously mentioned groups were divided into two subgroups. The first set of animals was anesthetized using intraperitoneal injection of urethane (1.25 g/kg) and subjected to blood sampling, decapitation and cerebral tissue sampling. The second set of animals was subjected to induction of cerebral ischemic-reperfusion.

#### Induction of cerebral ischemic-reperfusion

After anesthesia, each rat was fixed on thermostatically controlled heating pad. A rectal thermometer was inserted and body temperature kept at 37°C and both common carotid arteries were exposed by a midline incision. Each carotid artery was freed from its adventitial sheath and vagus nerve, which was carefully separated and maintained. Ischemia was achieved by clamping the bilateral common carotid arteries for 30 min. Recirculation of blood flow was established by declamping and restoration of blood flow in the carotid arteries was confirmed by careful observation. Reperfusion was allowed for 60 min. [20]*.* All rats of (N), (D) and (D + Res) groups were subjected to the previous procedure except for clamping of carotid arteries and reperfusion.

At the end of this time, the rats were subjected to blood sampling, decapitation and cerebral tissue sampling. Blood was collected via Retro-orbital bleeding in a dry centrifuge tube, and centrifuged at 3000 rpm for 15 minutes for serum separation. Fresh serum samples were processed immediately for determination of glucose level. The remainder serum samples were stored as aliquots at -20°C for subsequent determination of triacylglycerol (TAG), total cholesterol (TC) and high density lipoprotein cholesterol (HDL-C).

Following blood collection, rats were killed by decapitation. The brain from each animal was removed, then either washed with 0.9% NaCl. and quickly frozen in liquid nitrogen (-169°C) for 5 minutes then stored at -20°C for further determination of lipid peroxidation in the form of MDA, GSH content, IL-4 level, as well as gene expressions of COX-2 or kept in 10% formalin solution.

### Biochemical assays in the serum

The following Parameters were measured in the serum using commercial kits provided by Spinreact, Co., Spain.: Glucose [21], triacylglycerol [22], cholesterol [23] and HDL-C [24]. Serum LDL-C was determined using the formula of *Friedewald et al.* [25]*.* Furthermore, atherogenic index was calculated according to the formula of *Sharma et al.* (atherogenic index = LDL-C/HDL-C) [26].

### Biochemical assays in cerebral tissues

Cerebral MDA was determined spectrophotometrically as a marker of lipid peroxidation by using thiobarbituric acid reagent, according to modified method of *Buege and Aust* [27]*.* GSH content was measured spectrophotometrically using Ellman’s reagent, according to the modified method of *Ahmed et al.* [28]*.* Also, cerebral IL-4 was determined according to *Nolan et al.* [29] by solid phase Enzyme Linked Immuno Sorbent Assay (ELISA) using rat IL-4 kit (RayBiotech, USA) and a microtiter plate reader capable of reading at 450 nm.

### Molecular biology assays in the cerebral tissues

#### RNA extraction

Total RNA was extracted from cerebral tissues using SV Total RNA isolation system (Promega, Madison, WI, USA) according to the manufacturer’s instructions.

#### Gene expression of COX-2

For amplification of target COX-2 gene, RT-PCR was run as 2 separate steps. Briefly, equal amounts of total RNA were reverse transcribed using the Moloney murine leukemia virus reverse transcriptase (Promega, Madison, Wisconsin), ribonuclease inhibitor (Promega), deoxynucleoside 5′ triphosphate and oligo-dT primer. The reaction was terminated by heating to 95 C for 10 minutes, followed by cooling to 4 C. The complementary DNA samples were amplified in the presence of Taq DNA polymerase (Promega), deoxynucleoside 5′ triphosphate, and the appropriate primer pairs (primers, annealing temperatures, number of PCR cycles and product sizes are listed in Table 1).

**Table 1:** **Sequence of primers used in the experiment**

Gene	Primer sequence	Annealing temp.	Number of PCR cycles	Product size
**Cox-2**	**Forward primer:**	**55°C**	**30**	**536 bp**
	5′-GCTTTCTCCAACCTCTCCTACTACA-3′			
	**Reverse primer:**			
	5′-CATGGGAGTTGGGCAGTCA-3′			
**Beta actin**	**Forward primer:**	**60°C**	**30**	**265 bp**
	5′-AACCCTAAGGCCAACCGTGAAA-3′			
	**Reverse primer:**			
	5′-TCATGAGGTAGTCTGTCAGGTC-3′			

#### Agarose gel electrophoresis

The PCR products were electrophoresed on 2% agarose gel, stained with ethidium bromide and visualized by UV transilluminator. It was performed using the gel documentation system (BioDO, Analyzer) supplied by Biometra (Gottingen, Germany).

### Histological studies

Cerebral tissues were kept in 10% formalin for at least 1 week, then dehydrated using ascending grades of ethyl alcohol (70%, 90% and 100%), then cleaned in xylene and embedded in paraffin. Cross sections of about 4 μm thickness were cut with a microtome, mounted on glass slides and stained with:

- Routine Hematoxylin and eosin (H&E) stain [30]*.* The sections were then examined under light microscope for histological changes.
- AgNOR stain [31]*.* AgNOR stained sections were examined under the light microscope then AgNOR dots were counted as brown dots in the nuclei of cells using 40× objective lens. 50 cells were studied in each case and the mean AgNOR per nucleus was calculated.
- Cresyl violet stain [32]*.* Neuronal quantification was done under 40× lens of light microscope. Ten high power fields from each group were randomly selected for cell quantification. The number of viable cells had lightly stained nuclei while dark stained neurons with shrunken cell bodies were excluded from quantification.

### Immunohistochemical studies

#### COX-2 Immunohistochemical reaction

The cerebral cortex sections were processed according to [33] using polyclonal COX-2 antibody (Thermo Scientific Pierce™ Catalog #: PA1-37505 , dilution 1:100). Then morphometric study was done to measure the optical density of COX-2 reaction in ten high power fields using a (Leica Qwin 500, England) image analyzer.

#### Bax (an apoptotic marker) Immunohistochemical reaction

The cerebral cortex sections were processed according to [34] using monoclonal Bax antibody (Thermo Fisher Scientific™ Catalog #: MA5-14003 , dilution 1:50). Then morphometric study was done to count the apoptotic cells to calculate cerebral apoptotic index (ratio of apoptotic cells to normal cells) and measure the optical density of Bax reaction. This study was carried out using a (Leica Qwin 500, England) image analyzer in 10 high power fields*.*

### Statistical analysis

All results were expressed as Mean ± SD. Statistical analysis was performed using SPSS program *(version 16; SPSS Inc., Chicago, Illinois, USA)*. Student “t” test and the analysis of variance (one way ANOVA) were used for comparison between groups [35] *.*

## Results

### Biochemical and hematologic parameters

Table 2 illustrates that administration of a single dose of STZ to normal adult male albino rats induced diabetes as indexed by a significant increase (three folds) in serum glucose level, dyslipidemia as shown by a significant elevation of serum TAG, TC, serum LDL-C and atherogenic index and a significant decrease in serum HDL-C and finally a significant increase in cerebral MDA and depletion of cerebral GSH content as compared to normal rats. Also, cerebral ischemic-reperfusion in diabetic rats resulted in a significant increase in cerebral MDA content and a significant depletion of cerebral GSH in comparison to the diabetic group (D).

**Table 2:** **Effect of resveratrol on serum glucose, oxidative stress markers and lipid profile in studied groups**

	N	D	D + Res	DIR	DIR + Res
**Serum glucose (mg/dl)**	103.3 ± 8.2	341.8 ± 21.42*	241.3 ± 10.63^a^	348.5 ± 10.8*	261.7 ± 14.83^b^
**Cerebral MDA (nmol/g.tissue)**	178.8 ± 14.18	442.0 ± 17.05*	271.8 ± 29.4^a^	974.8 ± 45.19*^#^	684.8 ± 51.16^b^
**Cerebral GSH (nmol/g.tissue)**	320.5 ± 26.73	141.2 ± 17.79*	234.7 ± 12.42^a^	100.7 ± 13.8*^#^	226.3 ± 9.81^b^
**Serum TAG (mg/dl)**	72.5 ± 6.95	204.8 ± 14.66*	121.3 ± 10.75^a^	220.5 ± 12.34*	127 ± 11.25^b^
**Serum TC (mg/dl)**	77.83 ± 6.62	164.7 ± 14.58*	107.8 ± 10.17^a^	168.3 ± 14.42*	111.9 ± 10.54^b^
**Serum HDL-C (mg/dl)**	26.67 ± 2.07	17 ± 1.52*	22.17 ± 1.6^a^	16.02 ± 1.44*	20.87 ± 1.48^b^
**Serum LDL-C (mg/dl)**	36.67 ± 4.68	106.7 ± 12.29*	61.4 ± 9.78^a^	108.2 ± 12.81*	65.62 ± 10.06^b^
**Atherogenic index**	1.37 ± 0.21	6.33 ± 1.11*	2.77 ± 0.55^a^	6.83 ± 1.25*	3.18 ± 0.57^b^

All results were expressed as mean ± SD, (n = 6).

*Significantly different from N at P < 0.01.

^#^Significantly different from D at P < 0.01.

^a^Significantly different from D at P < 0.05.

^b^Significantly different from DIR at P < 0.05.

N, D, D + Res, DIR, DIR + Res represent control, diabetic, diabetic treated with resveratrol, diabetic with cerebral ischemic-reperfusion, diabetic with cerebral ischemic-reperfusion treated with resveratrol groups.

Treatment of the diabetic group and the diabetic group with cerebral ischemic-reperfusion with resveratrol for six weeks resulted in a significant decrease in serum glucose level, serum TAG, TC, serum LDL-C, atherogenic index and cerebral MDA and a significant increase in serum HDL-C and cerebral GSH content when compared to the corresponding control groups.

### Effect of resveratrol on cerebral IL-4

**Figure 1:**
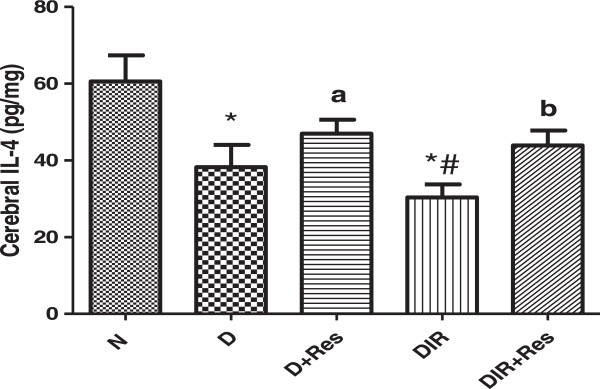
**Effect of resveratrol on cerebral IL-4 content in studied groups.** (*) Significantly different from N at P < 0.01. (#) Significantly different from D at P < 0.01. (a) Significantly different from D at P < 0.05. (b) Significantly different from DIR at P < 0.05.

Diabetic rats and diabetic rats with cerebral ischemic-reperfusion showed significant decrease in cerebral IL-4 when compared to the normal control group. While treatment of these rats with resveratrol for six weeks resulted in a significant elevation of cerebral IL-4 in comparison to the corresponding control groups Figure 1.

### Effect of resveratrol on cerebral COX-2 gene expression

**Figure 2:**
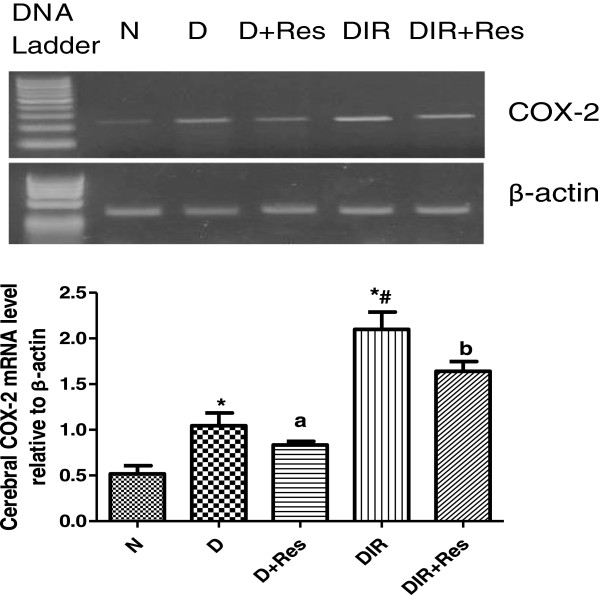
**Effect of resveratrol on relative cerebral COX-2 gene expression (COX-2/β-actin) in studied groups.** An agarose gel electrophoresis showed PCR products of mRNA gene expression level of COX-2 gene and normal control gene (β-actin). Lane DNA ladder: 100pb DNA marker, lanes N, D, D + Res, DIR, DIR + Res represent control, diabetic, diabetic treated with resveratrol, diabetic with cerebral ischemic-reperfusion, diabetic with cerebral ischemic-reperfusion treated with resveratrol groups. (*) Significantly different from N at P < 0.01. (#) Significantly different from D at P < 0.01. (a) Significantly different from D at P < 0.05. (b) Significantly different from DIR at P < 0.05.

The diabetic group and the diabetic group with cerebral ischemic-reperfusion showed a significant upregulation of cerebral COX-2 gene expression while treatment of these groups with resveratrol for six weeks led to significant downregulation of cerebral COX-2 gene expression in comparison to the corresponding control groups Figure 2.

### Histological results

**Figure 3:**
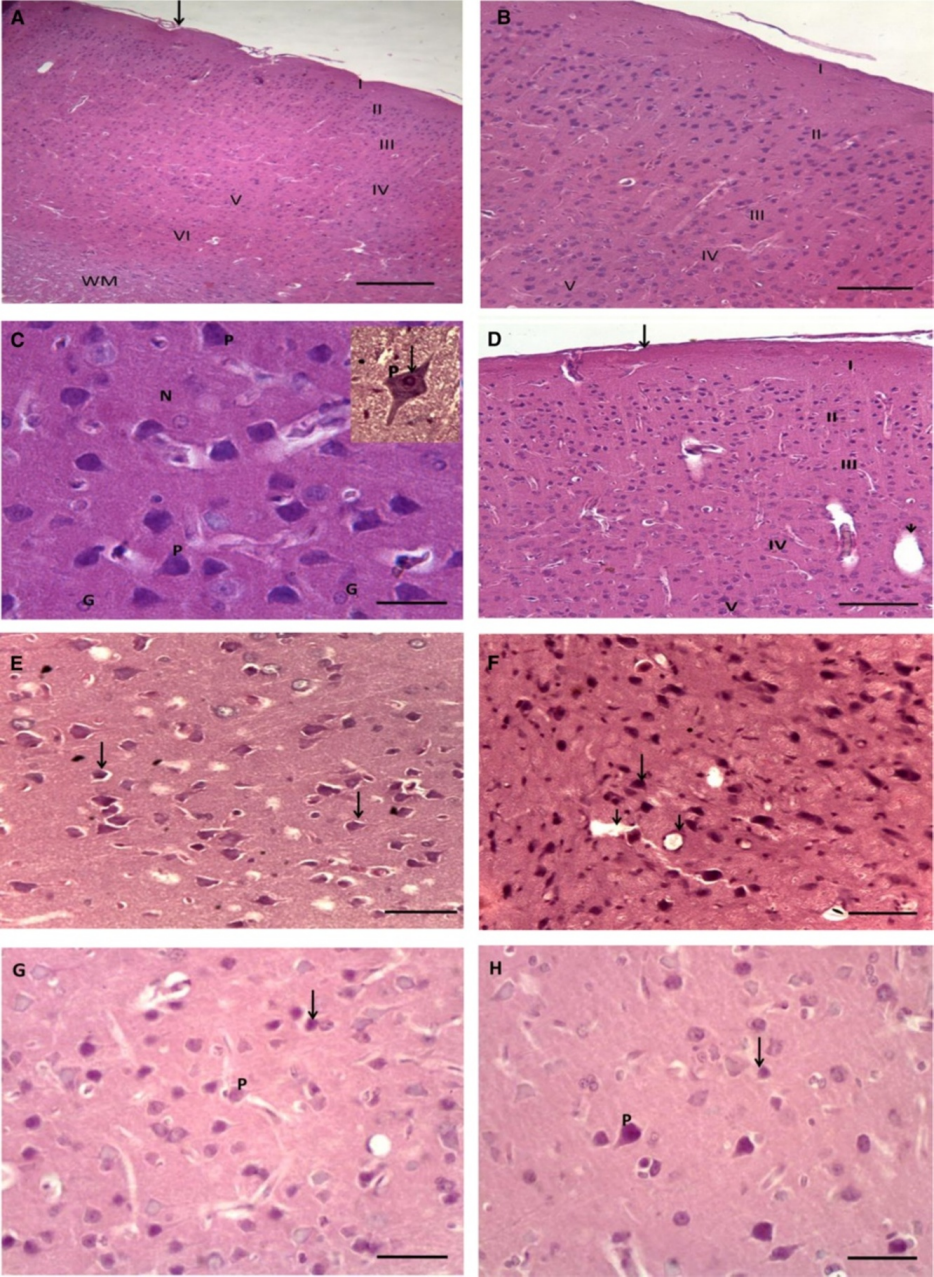
**Photomicrographs of sections in the cerebral cortex of adult male albino rats. (A)** control group showing pia matter containing blood vessel (arrow). Six layers are identified in the cerebral cortex; outer molecular layer (I), external granular layer (II), external pyramidal layer (III), inner granular layer (IV), inner pyramidal (V), and the polymorphic layer (VI). Also, white matter can be seen. **(B)** higher magnification of the previous section. **(C)** control group showing normal pyramidal cell (P). This cell has multipolar shape with basophilic cytoplasm and large, rounded vesicular nucleus (arrow). Granular cells (G) can be seen with large open face nuclei, prominent nucleolus and little cytoplasm. The pink-stained background is the neuropil (N) **(D)** diabetic group showing pia matter (arrow), dilated blood vessel (short arrow). **(E)** diabetic group showing eosinophilic degeneration in pyramidal cells, noticed by contracted cells with loss of cell processes, eosinophilic cytoplasm and small darkly stained nucleus (arrow). Some cells are surrounded with halos **(F)** diabetic cerebral ischemic-reperfused group showing eosinophilic degeneration in all pyramidal cells, noticed by shrunken cells with loss of their processes, eosinophilic cytoplasm and small darkly stained nucleus (arrow). Some cells are surrounded with halos. Many congested blood vessels can be noticed (short arrows). **(G)** diabetic group treated with resveratrol showing many pyramidal cells with normal shape (P). However, some cells still show features of eosinophilic degeneration (arrow). **(H)** diabetic cerebral ischemic-reperfused group treated with resveratrol showing the similar results as in diabetic group treated with resveratrol. [A → H&Ex40 (scale bar represents 100 μm), B, D → H&Ex100 (scale bars represent 40 μm), C, E, F, G, H → H&E stain x400 (scale bars represent 10 μm)].

In H&E-stained sections, the cerebral cortex of the normal control group was covered by pia matter containing blood vessels. Six layers were identified in the cerebral cortex; outer molecular layer, external granular layer, external pyramidal layer, inner granular layer, inner pyramidal, and the polymorphic layer. The molecular layer was thick and contained dense plexus of nerve fibers with few cells. Whereas, the external granular and external pyramidal contained numerous granular cells and pyramidal cells. While the internal granular and internal pyramidal showed few granular cells and pyramidal cells. The pyramidal cell had multipolar shape with basophilic cytoplasm and large, rounded vesicular nucleus. Granular cells could be seen with large open face nuclei, prominent nucleolus and little cytoplasm. The pink-stained background was the neuropil Figure 3-A, B, C.Light microscope examination of H&E stained sections of cerebral cortex of the diabetic and diabetic cerebral ischemic-reperfused groups showed a picture of eosinophilic degeneration of pyramidal cells. The cells appeared contracted, lost their processes with eosinophilic cytoplasm and small, darkly stained nuclei. Some neurons; surrounded by halos could be observed Figure 3-D, E, F. On the other hand, treatment of diabetic rats and diabetic cerebral ischemic-reperfused rats with resveratrol resulted in moderate improvement of cerebral tissue. Resveratrol restored the normal architecture of cerebral tissue to some extent. Some of cells appeared normal in shape however, other cells showed a picture of eosinophilic degeneration, Figure 3-G, H.

**Figure 4:**
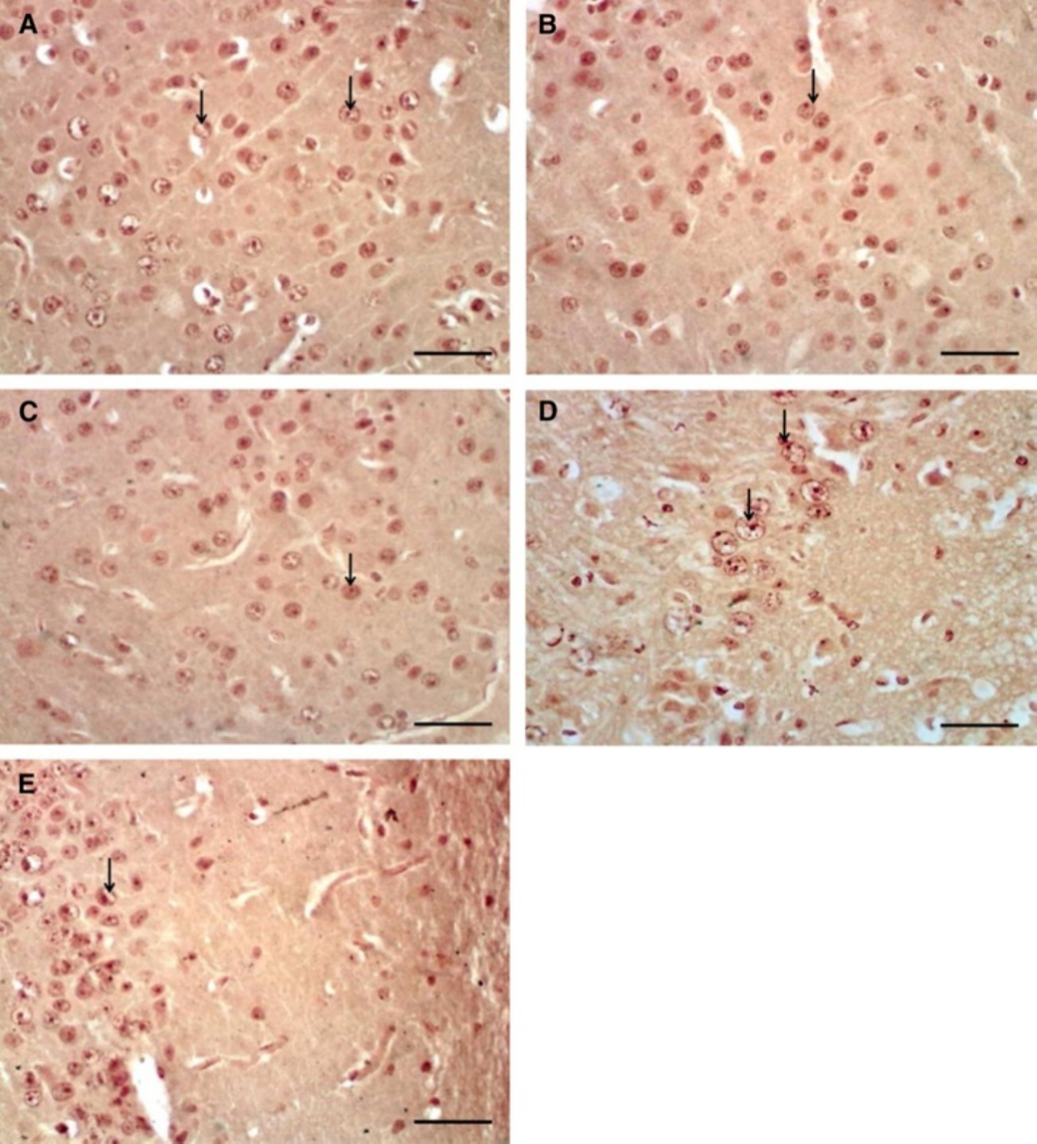
**Photomicrographs of sections in the cerebral cortex of adult male albino rats. (A)** control group **(B)** diabetic group **(C)** diabetic cerebral ischemic-reperfused group **(D)** diabetic group treated with resveratrol **(E)** diabetic cerebral ischemic-reperfused group treated with resveratrol. All these micrographs showing AgNOR dots (arrows) in the nuclei of the neuronal cells [A, B, C, D, E AgNOR stain x400 (scale bars represent 10 μm)].

Examination of cerebral cortex sections stained with AgNOR stain revealed that AgNOR positive stain appeared as brown dots in pale yellow stained nuclei. The mean AgNOR counts were significantly low in the diabetic and diabetic ischemic-reperfused groups when compared to the normal control group. In the diabetic and diabetic ischemic-reperfused groups treated with resveratrol, mean AgNOR counts showed a moderate increase (but not significant) when compared to the diabetic and diabetic ischemic-reperfused groups Figure 4 and Table 3.

**Table 3:** **Effect of resveratrol on AgNOR, cresyl violet and mean optical density of COX-2 immunohistochemical reaction**

	N	D	D + Res	DIR	DIR + Res
**Mean numbers of AgNOR dots in the nuclei of 50 cells**	0.76 ± 0.83	0.38 ± 0.68*	0.52 ± 0.78	0.32 ± 0.61*	0.48 ± 0.67
**Mean number of viable cells stained with cresyl violet**	23.3 ± 3.39	13.6 ± 6.34	17 ± 4.54	9.3 ± 1.24*	14.3 ± 2.86^b^
**Mean of COX-2 optical density**	9.02 ± 2.45	21.54 ± 2.95*	13.03 ± 2.34^a^	32.06 ± 3.74*^#^	19.34 ± 4.25^b^

All results were expressed as mean ± SD, (n = 6).

*Significantly different from N at P < 0.05.

^#^Significantly different from D at P < 0.05.

^a^Significantly different from D at P < 0.05.

^b^Significantly different from DIR at P < 0.05.

N, D, D + Res, DIR, DIR + Res represent control, diabetic, diabetic treated with resveratrol, diabetic with cerebral ischemic-reperfusion, diabetic with cerebral ischemic-reperfusion treated with resveratrol groups.

**Figure 5:**
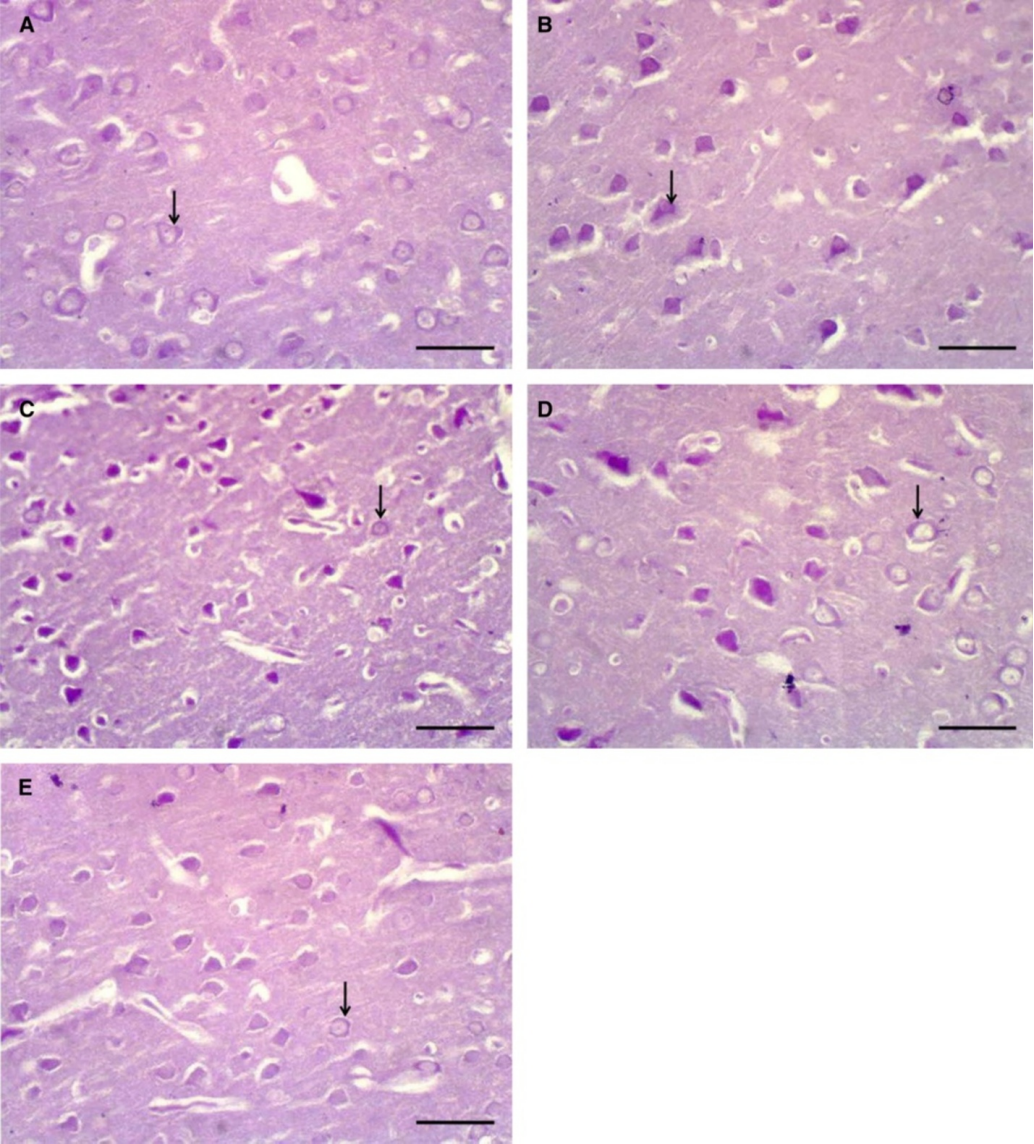
**Photomicrographs of sections in the cerebral cortex of adult male albino rats. (A)** control group **(B)** diabetic group **(C)** diabetic cerebral ischemic-reperfused group **(D)** diabetic group treated with resveratrol **(E)** diabetic cerebral ischemic-reperfused group treated with resveratrol. All these micrographs showing surviving neurons (arrows). Viable neurons have lightly stained nuclei while darkly stained neurons with shrunken cell bodies were excluded from quantification. [A, B, C, D, E cresyl violet stain x400 (scale bars represent 10 μm)].

**Figure 6:**
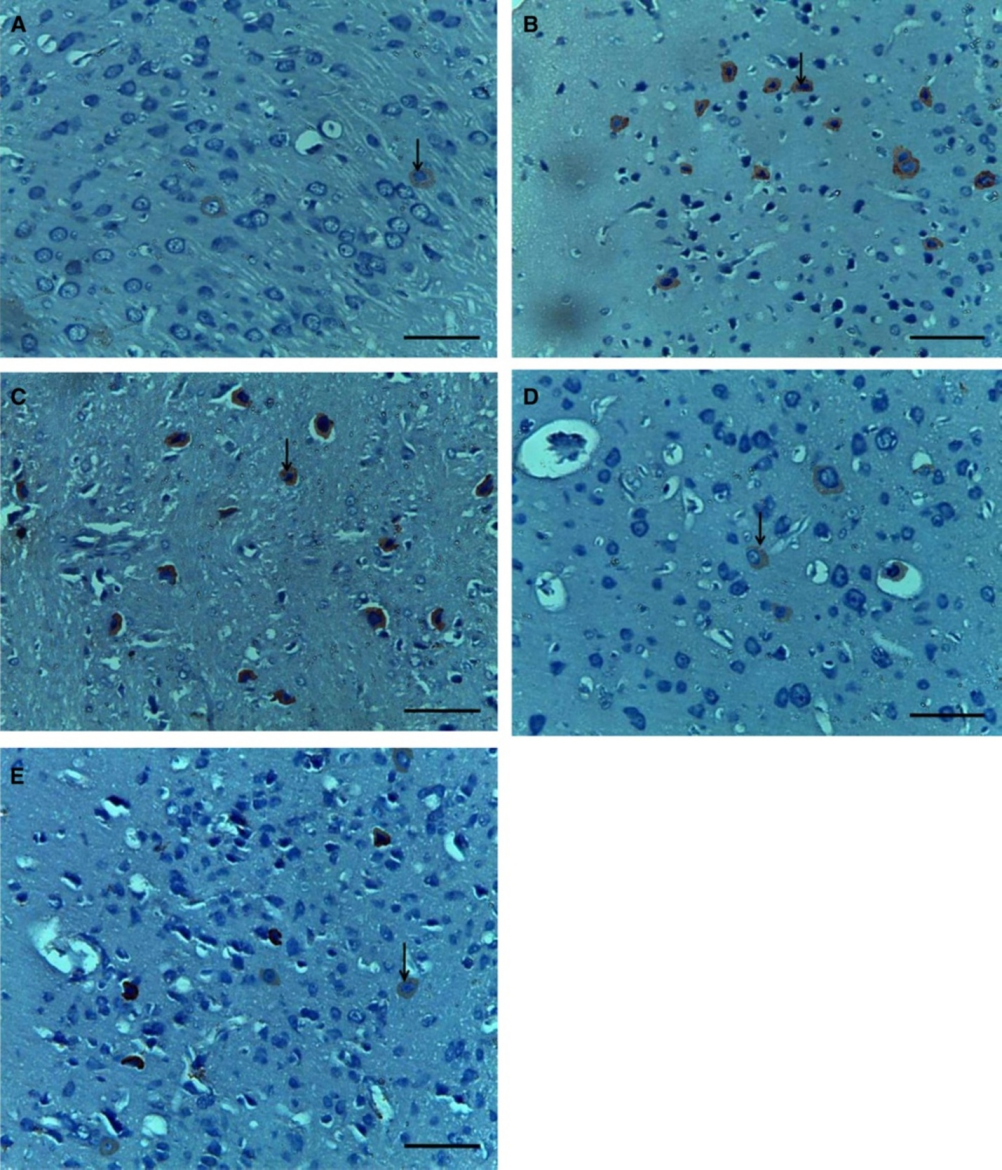
**Photomicrographs of sections in the cerebral cortex of adult male albino rats. (A)** control group showing weak positive immunoreaction for COX-2 (arrow). **(B)** diabetic group showing strong positive immunoreaction for COX-2 (arrow). **(C)** diabetic cerebral ischemic-reperfused group showing strong positive immunoreaction for COX-2 (arrow). **(D)** diabetic group treated with resveratrol showing moderate immunoreaction for COX-2 (arrow). **(E)** diabetic cerebral ischemic-reperfused group treated with resveratrol showing moderate immunoreaction for COX-2 (arrow). [A, B, C, D, E using Avidin-biotin peroxidase stain with Hx counter stain x400 (scale bars represent 10 μm)].

Microscopic examination of cerebral cortex sections stained with cresyl violet stain of the studied groups showed pathological changes. Upon quantification of viable cells in cerebral cortex, the diabetic and diabetic ischemic-reperfused groups exhibited decrease (not significant in the diabetic group and significant in the diabetic ischemic-reperfused group) in the mean number of surviving neurons when compared to the normal control group. While the treated groups with resveratrol revealed a moderate increase (not significant in the diabetic treated group and significant in the diabetic ischemic-reperfused treated group) in the mean number of surviving neurons Figure 5 and Table 3.COX-2 immunoexpression appeared as brown cytoplasmic reaction. Immunohistochemical examination of cerebral cortex sections of the normal control group showed weak positive immunoreactions for COX-2 Figure 6-A. The diabetic and diabetic ischemic-reperfused groups showed strong positive immunoreaction for COX-2, Figure 6-B, C. On the other hand, the diabetic and diabetic ischemic-reperfused groups treated with resveratrol revealed moderate immunoreaction for COX-2, Figure 6-D, E.

**Figure 7:**
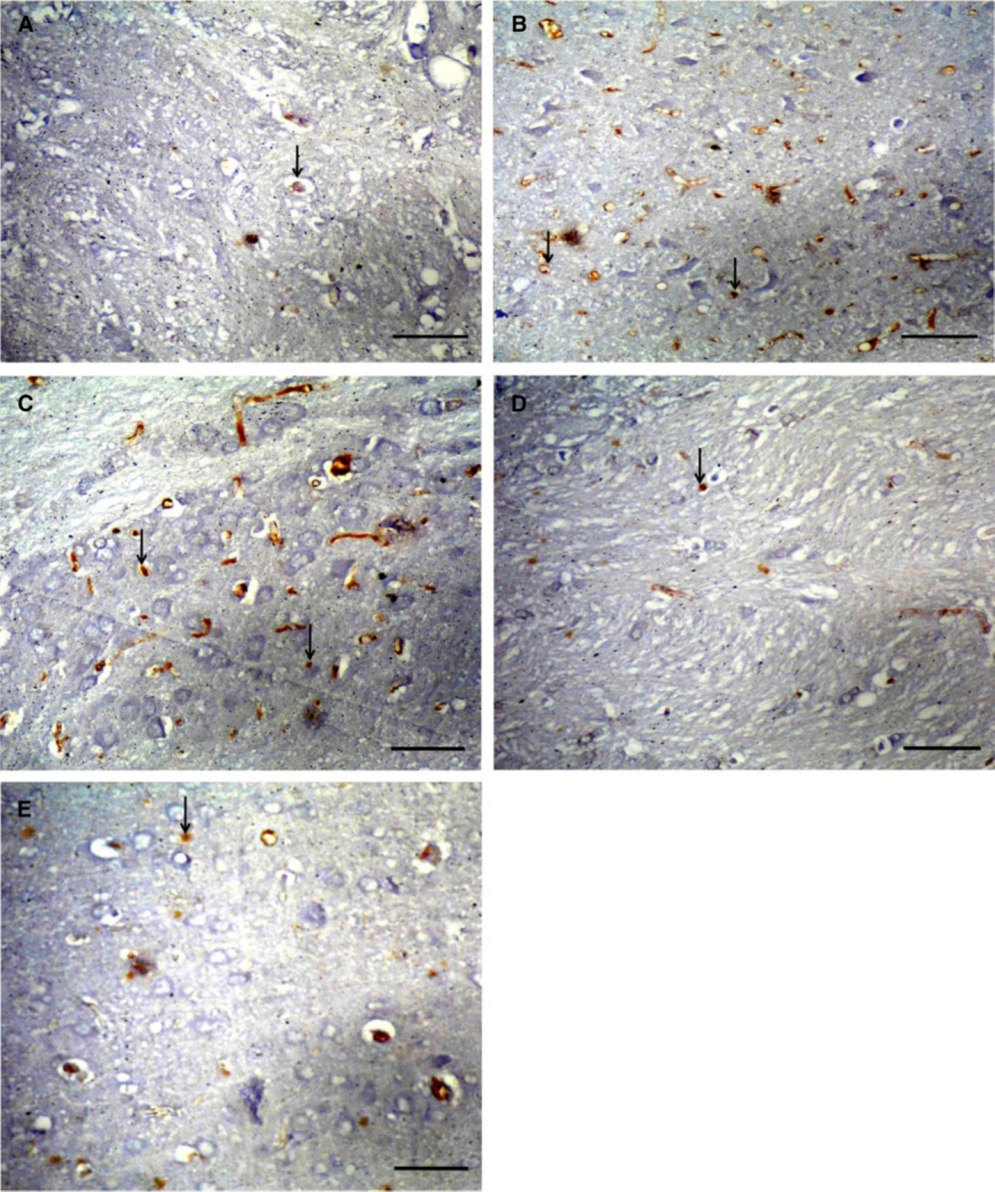
**Photomicrographs of sections in the cerebral cortex of adult male albino rats. (A)** control group showing weak positive immunoreaction for Bax (arrow). **(B)** diabetic group showing strong positive immunoreaction for Bax (arrows). **(C)** diabetic cerebral ischemic-reperfused group showing strong positive immunoreaction for Bax (arrows). **(D)** diabetic group treated with resveratrol showing moderate immunoreaction for Bax (arrows). **(E)** diabetic cerebral ischemic-reperfused group treated with resveratrol showing moderate immunoreaction for Bax (arrows). [A, B, C, D, E using Avidin-biotin peroxidase stain with Hx counter stain x400 (scale bars represent 10 μm)].

Table 3 showed significant increase in the mean of the optical density of COX-2 immunoreaction in the diabetic and diabetic cerebral ischemic-reperfused groups in comparison to the normal control group. On the other, treatment of the diabetic and diabetic cerebral ischemic-reperfused groups with resveratrol resulted in a significant reduction in the mean of optical density of COX-2 immunoreaction in comparison to the corresponding control groups.Immunohistochemical examination of cerebral cortex sections of the normal control group showed weak positive immunoexpression for Bax, Figure 7-A. However, cerebral cortex of the diabetic and diabetic ischemic-reperfused groups showed strong positive immunoreaction for Bax, Figure 7-B, C. Conversely, immunohistochemical examination of the cerebral cortex of the diabetic and diabetic ischemic-reperfused groups treated with resveratrol revealed moderate immunoreaction for Bax, Figure 7-D, E.

Table 4 showed significant cerebral apoptosis measured by a significant increase in cerberal apoptotic index (ratio of apoptotic cells to normal cells) and the mean optical density of Bax immunoreaction in the diabetic and diabetic cerebral ischemic-reperfused groups in comparison to the normal control group. On the other hand, treatment of the diabetic and diabetic cerebral ischemic-reperfused groups with resveratrol resulted in a significant reduction of cerberal apoptotic index and the mean optical density of Bax immunoreaction in comparison to the corresponding control groups.

**Table 4:** **Effect of resveratrol on apoptotic markers (cerebral apoptotic index and optical density mean of BAX immunohistochemical reaction)**

	N	D	D + Res	DIR	DIR + Res
**Cerebral apoptotic index**	0.1 ± 0.01	0.91 ± 0.13*	0.48 ± 0.1^a^	1.22 ± 0.12*^#^	0.52 ± 0.08^b^
**Optical density mean of BAX reaction**	55.4 ± 6.33	111.8 ± 13.13*	79.71 ± 7.16^a^	142.4 ± 15.31*^#^	83.74 ± 7.53^b^

All results were expressed as mean ± SD, (n = 6).

*Significantly different from N at P < 0.01.

^#^Significantly different from D at P < 0.01.

^a^Significantly different from D at P < 0.05.

^b^Significantly different from DIR at P < 0.05.

N, D, D + Res, DIR, DIR + Res represent control, diabetic, diabetic treated with resveratrol, diabetic with cerebral ischemic-reperfusion, diabetic with cerebral ischemic-reperfusion treated with resveratrol groups.

## Discussion

Administration of STZ (45 mg/kg) in adult male albino rats resulted in induction of diabetes that was confirmed by a remarkable increase in serum glucose level as compared to normal rats. Other symptoms such as weight loss, polyuria, polydepsia and polyphagia were also observed in diabetic rats (data not reported). These results are in agreement with other studies [1, 2, 10, 36] *.* Moreover, marked dyslipidemia, oxidative stress and inflammatory responses were observed in diabetic rats and these findings are in harmony with various reported studies [2, 36–39] *.*

In the present study, diabetic rats showed a picture of eosinophilic degeneration and strong Bax immunoreaction of pyramidal cells that were contracted with eosinophilic cytoplasm, small darkly stained nuclei and some of them were surrounded with halos and these findings are in agreement with *Amin et al.* [40]*.* These results were confirmed with AgNOR stain which showed a significant decrease in the mean number of AgNOR dots in the diabetic group. *Bhatt et al.* [41] stated that Nucleolar Organiser Regions (NORs) are segments of DNA, closely associated with nucleoli of the cells on the short arms of the acrocentric chromosomes, 13, 14, 15, 21 and 22, containing coding gene for Ribosomal RNA and contribute to the regulation of the cellular synthesis. Recent modification of a silver staining technique allows the interphasic NORs to be visualized under LM in conventional histopathological sections, where they are called as “Argyrophilic Nucleolar Organizer Regions (AgNORs)”. Also, cresyl violet stained sections of the diabetic group showed a decrease (not significant) in the mean number of viable neurons. *Pamidi et al.* [32] found that the diabetic rats exhibited a decrease in the mean number of surviving neurons, counted using cresyl violet stained sections of cerebral cortex, when compared to the control group. These results indicated a decrease in the activity of the neuronal cells of the cerebral cortex which is most probably due to oxidative stress and apoptosis. It has been reported that neurons in hyperglycemic environment displayed signs of apoptosis due to hyperglycemia-induced oxidative stress [4]*.* Also, *Zhao et al.* [42] proved that diabetes upregulated the expression of Bax and caspase-3 which led to apoptosis of the pyramidal neurons in STZ induced diabetic rats.

The current study showed that cerebral ischemic-reperfusion for diabetic rats specifically exaggerated oxidative stress, inflammation and apoptosis including a significant increase of cerebral content of MDA, upregulation of COX-2 gene expression, a severe depletion of cerebral GSH and IL-4 contents and a significant increase in the apoptotic index and the optical density of Bax reaction. The major pathological mechanisms of cerebral ischemic injury include excitotoxicity, oxidative stress, inflammation, and apoptosis, which are associated with mitochondrial dysfunction and a rapid decrease of adenosine triphosphate (ATP). Depletion of GSH in cerebral ischemia leads to lipid peroxidation and neuronal cell apoptosis, in which the Bcl-2 family proteins (e.g. anti-apoptotic Bcl-2, pro-apoptotic Bax) are involved [43, 44] *.* Furthermore, there is evidence that elevated ROS levels within mitochondria generated by cerebral ischemic-reperfusion alters the expression of pro-apoptotic factor Bax, anti-apoptotic Bcl-2 and caspase-3 [43, 45].

Histologically, the cerebral tissues of cerebral ischemic-reperfusion diabetic rats showed features of eosinophilic degeneration and some neurons were surrounded with halos. These findings are in agreement with *Levison Damr* [46] who stated that cerebral ischemia or anoxia led to eosinophilic degeneration, mostly of pyramidal cells of cerebral cortex as the whole cell shrinks, contracts, the cytoplasm loses its Nissl granules and becomes eosinophilic. The nucleus is basophilic, hyperchromatic, small and pyknotic and moves to more peripheral position and the nucleolus disappear. Also, AgNOR stained sections showed significant decrease in mean number of AgNOR dots. Furthermore, cresyl violet stain showed a significant decrease in the mean number of viable neurons. *Pamidi et al.* [32] supported our findings as they found that the untreated diabetes mellitus coupled with stress can induce highly significant damage in the neurons of rat cerebral which was shown by a significant decrease in the number of surviving neurons of cresyl violet stained sections.

In the present study, treatment of the diabetic and the diabetic ischemic-reperfused rats with resveratrol induced a remarkable reduction of plasma glucose level and corrected the diabetic dyslipidemia. These results are in harmony with other studies [16, 47] which reported that resveratrol reduced blood TAG, TC and LDL-C and elevated HDL-C in hypercholesterolemic rats*.* Moreover, *Gnoni and Paglialonga* [48] reported that resveratrol decreased fatty acid and TAG synthesis through inhibition of fatty acid synthase in isolated rat hepatocytes. This may represent a potential mechanism contributing to the reported hypolipidemic effect of resveratrol.

Administration of resveratrol significantly ameliorated diabetes-induced oxidative stress, inflammation and apoptosis. Various studies suggested the neuroprotective activity of resveratrol through its antioxidant and anti-inflammatory properities [15, 49, 50]. Resveratrol was reported to inhibit lipid peroxidation and neuronal cell death induced by oxidative stress and enhance various antioxidant enzymes [10, 51]*.* These effects could be attributed to its property as a potent scavenger of ROS and RNS.

*Zhang et al.* [49] demonstrated interesting anti-inflammatory activities for resveratrol. It can attenuate the activation of immune cells and the subsequent synthesis and release of pro-inflammatory mediators through the inhibition of the transcriptional factors such as NF-κB. In addition, it has been shown to inhibit the activation of microglia, cerebral microphages and reduce the production of pro-inflammatory mediators. Therefore, resveratrol may exert neuroprotection in neurodegenerative diseases accompanied by microglial activation. This hypothesis is best evidenced with the present study showing that resveratrol increased cerebral IL-4, anti-inflammatory cytokine targeting the microglia, in treated animals.

IL-4 was reported to suppress NF-κB which is a transcription factor that resides in the cytoplasm of every cell and translocates to the nucleus when activated. Its activation is induced by a wide variety of agents including stress, inflammatory stimuli and free radicals. Activation of NF-kB upregulates the expression of COX-2 (an inflammatory enzyme) [52]*.* The present study reported a significant upregulation of COX-2 gene expression in cerebral cortex of diabetic and diabetic cerebral ischemic-reperfused rats while resveratrol treatment downregulated COX-2 gene expression of treated animals. These results are in accordance with *Kumar and Sharma* [18] who stated that COX-2 enzyme is an inducible enzyme, becoming abundant in activated macrophages and other cells at sites of inflammation. This enzyme has been reported to be elevated in metabolic diseases as well as in diabetic condition.

The present study showed that treatment of the diabetic and the diabetic ischemic-reperfused groups with resveratrol reduced the extent of eosinophilic degeneration and apoptosis of neurons and showed moderate improvement of the cerebral tissues. Also, the mean number of AgNOR dots showed an increase (not significant) and the mean number of viable neurons stained with cresyl violet stain showed an increase (significant in the diabetic ischemic-reperfused group and not significant in the diabetic group) upon treatment with resveratrol. Therefore, these results showed that resveratrol has anti-apoptotic potency in consistent with previous reports [44, 53, 54]*.* Also, [55] reported that resveratrol treatment attenuated rat cerebral damage after cerebral ischemia by downregulation of Bax expression.

The findings of the present study introduced new insights into the pathogenesis and treatment of neurodegenerative diseases, especially diabetic cerebral complications.

## Conclusion

In conclusion, beside antioxidant properties of resveratrol, it exerted beneficial hypoglycemic, hypolipidemic and anti-inflammatory effects regarding diabetes-induced cerebral complications in diabetic and diabetic cerebral ischemic-reperfused rats. These effects suggest resveratrol as a promising neuroprotective agent in diabetes-induced cerebral complications. Therefore, this study recommended such drug in diabetic complications especially neuropathy to limit the risks of cerebral complications.
